# ‘Leading by Leveraging Situations’: A Participant Observation Study Exploring How Registered Nurses Lead Close to Older Adults in Municipal Home Healthcare

**DOI:** 10.1111/opn.70102

**Published:** 2026-10-25

**Authors:** Therese Stien, Lisa Herulf Scholander, Lise‐Lotte Jonasson, Karin Josefsson

**Affiliations:** ^1^ Faculty for Nursing and Health Science Nord University Bodø Norway; ^2^ Department of Neurobiology, Care Sciences and Society Karolinska Institutet Huddinge Sweden; ^3^ Research and Development Unit for Older Persons (FOU nu) Stockholm Health Care Services Järfälla Sweden; ^4^ Department of Nursing Science, School of Health and Welfare Jönköping University Jönköping Sweden

**Keywords:** home healthcare, home nursing, leadership, older adults, participant observations, reflexive thematic analysis, registered nurses

## Abstract

**Background:**

Leadership plays a crucial role in the quality of care and patient safety, especially in the context of strained resources. Municipal home healthcare is facing challenges, for example, fewer care workers to meet increasing care needs. Registered nurses are key figures in municipal home healthcare, and they are leaders in the direct care of older adults. There is a lack of knowledge of how registered nurses lead close to older adults. The aim of this study was to explore how registered nurses lead close to older adults in municipal home healthcare.

**Methods:**

Eight (*n* = 8) registered nurses participated in 50 h of participant observations. Meetings between registered nurses and older adults, and the time between these interactions, were observed to explore how the registered nurses lead close to older adults. Fieldnotes from observations and informal talks with the registered nurses were collected and analysed via reflexive thematic analysis.

**Findings:**

The leadership of registered nurses close to older adults was portrayed into three themes: (1) ‘starting from the older adults’ implies viewing the older adults holistically and prioritising their needs over the needs of the organisational system; (2) ‘adapting to the particular’, which entails tailoring the environment and themselves to the unique in each situation: and (3) ‘utilising professional knowledge’ to both prepare their leadership and to lead. These three themes support an overarching theme showing how registered nurses lead close to older adults by ‘leveraging situations’ encountered in their work.

**Conclusion:**

This study provides insights into registered nurses' leadership close to older adults in home healthcare. The participant observations revealed that this leadership entails leveraging situations within the organisational framework, implying a need for registered nurses' flexibility and the ability to adapt to the prerequisites in this sector to provide good and safe care. These findings indicate it is time for organisations to align care for older adults and those who provide and lead the care. The findings illustrate how registered nurses enact leadership close to older adults through situated and adaptive practices in direct nursing care. The adaptive leadership practices of registered nurses might impact the quality of care. Providing registered nurses with the prerequisites to lead close to older adults, through means such as guidelines and educational efforts, might contribute to the long‐term sustainability of municipal home healthcare.

**Implications for Practice:**

Insights gained from this study indicate that RNs' leadership in HCC is performed by leveraging situations in their workday, which might be disguising potential consequences of a task‐focused organization. This could affect the quality of care and RNs' work satisfaction. Subsequently, this study might affect the sustainability of HHC as a growing sector. Future research should explore how leadership contributes to sustainable development by balancing quality of care, health economic considerations and environmental impacts. RNs can apply the findings from this study to recognize and gain valuable insights in their own work. Decision‐makers and educators should recognize how leadership is performed by RNs close to older adults. Facilitating educational efforts might advance RNs' leadership skills and competence, thereby enhancing the quality of care and contributing to the long‐term sustainability of this growing sector in the future.

## Summary

**What does this research add to existing knowledge in gerontology?:**

- Registered nurses close to older adults enact leadership at the micro level in municipal home healthcare by starting out from the older adults, adapting to the particular and utilising professional knowledge to leverage situations.
- This highlights how leadership close to older adults is embedded in day‐to‐day clinical practices and shaped by contexts.

**What are the implications of this new knowledge for nursing care for and with older adults?:**

- Registered nurses' leadership is performed by leveraging situations in their workday, which might disguise potential consequences of a task‐focused organisation.

**How could the findings be used to influence practice, education, research, and policy?:**

- Decision makers, for example, municipal directors and nurse educators, should recognise how leadership is performed by registered nurses close to older adults and facilitate educational efforts to advance registered nurses' leadership skills and competence.
- The municipal healthcare organisations and policymakers should implement structural measures to avoid placing the responsibility of holistic care on the individual registered nurse; it should be embedded in the organisational framework.

## 1 Introduction

In Norway, home healthcare (HHC) is publicly funded and organised by municipalities (Vabø 2012). HHC services are allocated through individualised care contracts with specified time frames. The provision and administration of HHC services are often divided into two separate units, known as the purchaser–provider model (Holm et al. 2017; Eide et al. 2022). One part decides what kind of help is needed, and another part provides the help. HHC serves the entire population, but most of the patients are older adults (Statistics Norway 2024), aged 65 years or older (The World Health Organization 2024). Registered nurses (RNs) who work in HHC provide care for patients with unique and complex needs (Fjørtoft et al. 2021). HHC is a distinct form of healthcare that differs from other settings in terms of competencies, needs, safety barriers and patient–nurse relationships (Fjørtoft 2016; McGarry 2010; Shahrestanaki et al. 2023; Neal 1999).

The concept of RNs' leadership lacks consistency in the literature and there are several different descriptions, such as clinical, bedside, informal or first‐line leadership (Downey et al. 2011; Stanley et al. 2022; Ingstad 2021). Furthermore, literature can be unclear regarding who is performing leadership. RNs do not always consider themselves leaders (Josefsson and Hansson 2011), possibly because they equate leadership with formal authority or some sort of connecting title (Clark 2008). RNs leading close to older adults have been described as ‘multi‐artists’, demonstrating skills such as trust‐building, exercising control and maintaining competence (Claesson et al. 2020). Seventy per cent of RNs report facing challenges when leading close to older adults (Lillsjö et al. 2023). Leadership among RNs who provide direct care to patients has been described as ‘the hidden treasure’ (Downey et al. 2011). RNs' leadership affects both care quality and patient safety (Wong et al. 2013; Murray and Cope 2021).

Leadership is recognised as a process in which a group is influenced by an individual to achieve a shared goal (Northouse 2022). Leadership and management share similarities and are often parallel processes; nevertheless, they are not the same (Northouse 2022). Management implies ways to control and maintain effectiveness (Northouse 2022). Leadership is person‐oriented and relational, and leaders utilise themselves in relation to others to influence others' effort and adaptability (Ladegård and Vabo 2010).

In this study, RNs' leadership is leadership enacted by RNs close to older adults, their next of kin and the care staff in direct nursing care (Lillsjö et al. 2023; Claesson et al. 2021). This definition does not include management, reflecting the inherent differences between the processes.

## 2 Aim

The aim of this study was to explore how registered nurses lead close to older adults in municipal home healthcare.

## 3 Methods

### 3.1 Design and Methodology

This was an explorative, inductive qualitative study grounded in interpretive traditions (Delanty and Strydom 2003). Participant observations of RNs (*n* = 8) leading close to older adults in HHC were conducted (Roper and Shapira 2000; Spradley 1980; Hammersley and Atkinson 1995). To interpret context‐dependent content, a reflexive thematic analysis (RTA) was utilised (Braun and Clarke 2021, 2022).

### 3.2 Participants and Procedure

Eight RNs from three workplaces in HHC participated. The inclusion criterion was a minimum of 1 year or more of work experience as a RN in HHC (Benner 1984). Managers in several HHC were contacted and provided an information letter, which managers referred to RNs meeting the criterion. To build trust and rapport, the observer met with the RNs a minimum of 1 day prior to observations. One meeting took place via telephone due to geographical distance. The participants had various levels of work experience in HHC, ranging from 1 to 35 years (mean = 14), and they were aged from 23 to 53 years old (mean = 39). Six female and two male RNs participated. The older adults in the meetings were 65 years and older and had the capacity to provide informed consent. Older adults were given information and recruited by the RNs and gave informed consent a minimum of 1 day prior to observation. One older adult was asked minutes prior to observation because of miscommunication between the observer and the RN. The next‐of‐kins and care staff present in the settings also provided informed consent. All participants were given an autonomous choice to participate. There was no prior relationship between the participants and the researchers.

### 3.3 Data Generation and Management

Fifty hours of observations were conducted from September to November 2024, by the first author. RNs were observed during their meetings with older adults and in between these interactions. The observations started with an open focus on how RNs lead and when something potentially interesting and relevant to the aim of the study emerged, the focus was narrowed to explore this aspect further (Roper and Shapira 2000). The research team acknowledges the active role the observer had in generating data (Braun and Clarke 2025). Consistent with RTA, this iterative shifting of focus occurred several times during data generation. Notes were taken as jottings during and/or after the meetings, depending on the situation. The notes were expanded into fieldnotes on the same day as the meetings or the day after (Roper and Shapira 2000). The data were generated in Norwegian and translated into English by the observing researcher during analysis.

Regarding the level of involvement in participant observation, the observer primarily took the role of participant‐as‐observer, but sometimes switched to observer‐as‐participant, depending on the situations (Roper and Shapira 2000). For example, when older adults experienced visual impairment, they increasingly sought the observer, which influenced the observer's participation in the meetings. Both day and evening shifts were observed. Most RNs were observed for 1 day and for an average of 5 h. Two participants were observed for 2 days. The duration of meetings between older adults and RNs varied from a few minutes to hours. Informal talks where RNs were asked to provide input on the observer's understanding of situations took place between meetings and after observations.

### 3.4 Analysis

Knowledge, meaning and leadership were viewed as contextually produced, relational and interpretive. RTA entails the flexibility needed to provide an analysis framework for a study with inductive and explorative aim, allowing for analysis to start when patterns of meaning or issues of potential interest could be identified (Braun and Clarke 2022). RTA recognises the importance of sensitivity in the researcher, while emphasising that preconceptions should not be allowed to direct the analytic process (Braun and Clarke 2022). The data were analysed inductively, and the themes represented important meanings related to the research topic; meaning themes were strongly linked to the data themselves and developed without trying to fit them in an existing coding frame or the researchers' analytic preconceptions (Braun and Clarke 2022). Themes captured meanings united by a central organising concept, interpreted beyond the semantic to the latent level (Braun and Clarke 2022).

The analysis was guided by the phases of RTA (Braun and Clarke 2022). In RTA, phases are distinct yet not linear steps; the analysis is a recursive process. The first phase is familiarisation. The second phase is generating initial codes. The third phase is searching for themes, the fourth phase reviews the themes and the fifth phase defines and names the themes. Phase six is producing the report. During the first phases of RTA, a question led the analysis: How do RNs lead in the HHC? Analysis started when patterns of meaning or issues of potential interest were identified (Braun and Clarke 2022) and in such, the first phase of analysis went somewhat parallel to the observations, guiding the observations to potential interest. Nevertheless, the entire dataset was read through several times at the end of the observations, and initial notes on how RNs lead were taken. In phase two, the dataset was coded systematically from start to end. Both semantic and latent codes were developed by identifying different meanings with different levels of abstraction. The codes were printed out, and in phase three, the codes were repeatedly clustered into subthemes by hand and linked to potential themes that carried central meaning, allowing for the exploration of various thematic possibilities. The potential themes were re‐evaluated, and a thematic map with themes and subthemes was developed and discussed by the research group in phase four. Themes were named based on interpretations aimed at concisely capturing the core meaning. In phase five, the themes became more defined, and two themes were merged because they shared a central meaning. In the last phase, when writing the report, a theme was interpreted as a result of other themes and classified as overarching.

### 3.5 Positionality

Participant observations are influenced by the observer's presence (Spradley 1980). The observer was an RN with previous work experience from municipal HHC. The observer thus inhabited close to a hybrid position, neither insider nor outsider, to the research field (Jootun et al. 2009). The insider effect of previously working as an RN in HHC facilitated assimilation to the field, with the outsider effect of previous work experiences being several years ago and now entering the HHC as an observer resulting in conscious reflexivity (Dwyer and Buckle 2009; Berger 2015).

### 3.6 Reflexivity

The authors strived to keep a reflexive approach, with mindful awareness of how research is shaped by researchers' positionality, choices and preunderstanding (Braun and Clarke 2021, 2022, 2019). During data generation, the observer kept a reflexive journal. Sensitivity and preunderstanding were discussed in depth within the research team before, during and after data generation and analysis, to avoid preunderstanding taking precedence over empirical data and to allow for mindful reflexivity in terms of how sensitivity influenced the analysis.

### 3.7 Rigour

The study's rigour rests on methodological coherence and trustworthiness (Lincoln and Guba 1985). To strengthen trustworthiness, the RNs in this study were from several different municipalities, differed in age and had varied relevant work experience, adding to the variation and thickness of data. This study followed the RTA approach from Braun and Clarke (Braun and Clarke 2022) as an iterative process acknowledging the subjective sensitivity and skills of the researchers while maintaining methodological coherence (Braun and Clarke 2021). Reflexive journaling and reflexive discussions within the research team enhanced rigour and provided insights on emerging interpretations. Through the flexible yet systematic data generation and analysis, transparent reporting and researcher reflexivity, rigour was strengthened.

### 3.8 Ethical Consideration

This study adheres to good research practices as outlined in the guidelines of NESH (the National Committee for Research Ethics in the Social Sciences and the Humanities (National Committee for Research Ethics in the Social Sciences and the Humanities 2023)) and the Helsinki declaration (World Medical Association 2024). The participants' identification and insured privacy were protected in line with the guidelines provided by the Norwegian Agency for Shared Services in Education and Research (SIKT), registered with D. no: 478281. This study from Nord University was assessed by the Regional Committee for Medical Research Ethics Northern Norway, REK North (D. no: 753491), and approval was waived according to national legislation, §2 and §9 of the Health Research Act (Act on Medical and Health Research 2008).

## 4 Findings

The findings showed how RNs lead close to older adults in HHC through three themes, supporting an overarching theme ‘leveraging situations’, see Figure 1.

**FIGURE 1:**
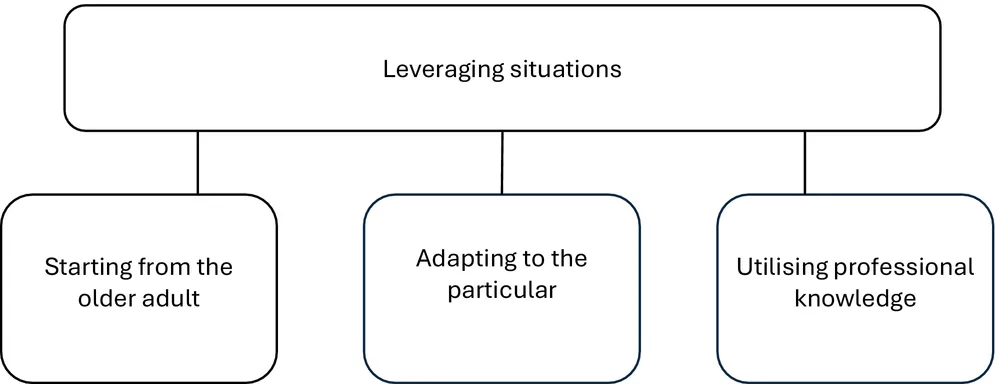
How registered nurses lead close to older adults in home healthcare is presented with an overarching theme and three themes.

### 4.1 Starting From the Older Adult

Starting from the older adult reflects how the RNs situated the older adults as a starting point for their leadership.

The different professional relationships in RNs' leadership close to older adults were centred around the older adult's interests and needs. Decisions regarding how and when to deliver care, changes in needs and cooperation with both older adults and their next‐of‐kin were made based on the older adult. A type of coordinating role originated from the older adult's needs. Examples of this role included contacting next‐of‐kin after acute events, providing explanations for changes or contacting other members of the multidisciplinary care team.

A characteristic of RNs' leadership in HHC was that the observed RNs focused on and prioritised the older adults' needs before what they referred to as ‘the system’. ‘The system’ described the framework and organisational structure that governed HHC. RNs provided care that was not approved or tweaked the approved care to fit older adults. RNs were leading by collaborating with older adults to make decisions on what to provide and the time needed. They assessed the needs and functions of older adults, sometimes on a day‐to‐day basis. Examples of this approach included using a longer time than scheduled, preparing meals or offering to assist in household chores such as washing up, feeding dogs or collecting mail when this was not on the work schedule. RNs used their professional discretion when needed, and had extended conversations instead of completing assigned tasks as administering feeding solutions if that was perceived as in the best interest of the older adult. When asked about how the RN influenced the provisions of care for the older adults, a participant said, ‘…the most important thing, for me, is providing the care my patients need, regardless of what is written here…’ pointing to the older adult's allocated decisions on a kind of spreadsheet.

By starting out from the older adults, the RNs' leadership could result in something more. An example of what appeared to be a simple and seemingly routine task that resulted in something more occurred when an RN took the older adult as a starting point at the end of an evening shift. The assigned task for this visit was removing compression treatment stockings. However, during the visit, the RN noticed that the personal alarm was not being worn and learned that the older adult avoided using it because it caused pain in her arm. The older adult lived alone and had nighttime trips to the bathroom, which placed her at risk of falling and being unable to summon help. The RN explored several alternatives, such as wearing the alarm on the opposite arm, attaching it to the walker or using a neck strap. Although the older adult agreed to some suggestions, the RN sensed that the solutions did not fully align with her daily practices. The RN emphasised finding a solution that would ensure a safer night‐time trip for the older adult. Rather than ending the visit after completing the assigned task, the RN continued exploring how to best meet the need. Together, they identified tripping hazards in the home and agreed on a temporary solution: removing loose rugs and leaving a light on at night. The RN interpreted the situation and led by finding a tailored solution for this older adult.

### 4.2 Adapting to the Particular

Adapting to the particular implied the ability to adapt leadership to each situation, both in meetings with older adults and to the framework of these meetings.

HHC takes place in the patient's home, wherever and no matter how these are situated. RNs actively worked together with older adults to make homes a suitable place for the older adult and the RNs. RNs moved furniture to create makeshift spaces needed for parenteral equipment or to provide safe environments, thereby reducing fall risk for older adults using walkers.

RNs led by adapting to the particular in tailoring their approaches. For example, RNs communicated differently depending on who they were communicating with. Several older adults had problems hearing, and RNs sat in front of them speaking politely, clearly and concisely. During home visits, RNs called older adults' doctors if the need was urgent. In this situation, the RNs acted like interpreters. To the physician, the language was formal and communication was like a report full of abbreviations and medical terms. When communicating with older adults and care staff, such as healthcare workers and nursing aides, the language was more informal and excluded most medical terms or abbreviations, although the topic was the same. The RNs ensured that everybody involved had the same information and was aligned, but the communication was adjusted to the receiver.

The RNs' workday was altered throughout the day due to various factors. RNs adapted their work to create a suitable, effective and practical workday. The recreation of a schedule was a continuous adaptation throughout the day, and RNs checked and tweaked their schedules several times. RNs could, for example, meet older adults who had acute conditions or abrupt changes in need. This resulted in RNs adapting their focus and intentions to the particular situations. Situations could require spending more time with one older adult, focusing on unplanned events, leaving less time for another older adult.

RNs leading by adapting to the particular went beyond merely adjusting their schedules. RNs modified behaviours to influence in achieving goals. An example of an RN adapting themselves and their behaviour was meeting an older adult who was bedridden with abdominal pain. In previous encounters with other older adults, this RN was notably animated, often laughing loudly, making jokes, and using expressive gestures that filled the room with energy. In this home, the RN's demeanour changed. The house had a general sense of neglect and carried a sharp unpleasant odour. Signs of physical and mental decline were clearly present; faeces were smeared down the side of the bed, and the bedroom felt confined and heavy. The older adult was reluctant to get out of bed and mostly responded to questions with a yes or no. Appearing withdrawn from both the surroundings and the interaction. The RN examined the older adult and found nothing new. The RN tried to get the older adult to come to the living room, but the attempts were met with refusal. The RN gently assisted the older adult into a sitting position in bed and began preparing breakfast. Despite the older adult's refusal to eat breakfast, the RN prepared a small meal and served it to the older adult in bed.

Smiling gently, the RN carefully adjusted both tone and body language to the situation, creating a warmer and more inviting atmosphere in a home otherwise marked by neglect and passivity. This situation required the RN to focus beyond nutrition or hygiene and adapt behaviour to inspire hope.

### 4.3 Utilising Professional Knowledge

The theme highlights how RNs utilised their professional knowledge in their leadership. RNs' professional knowledge is broad and covers several areas, such as communication, relationships, medical knowledge, gerontology and human behaviour. RNs used this knowledge to lead close to older adults. The duration of meetings between RNs and older adults ranged from a couple of minutes to hours. These meetings provided RNs with a snapshot of the older adult's functions, daily life and health, which RNs used to make assessments. RNs applied their professional knowledge to this snapshot to seek interventions or solutions that matched care needs in both acute and more ongoing stable settings.

RNs also utilised their professional knowledge to prepare their leadership. For example, RNs read patient reports before entering the home to obtain information about the older adult and situation. One RN sat in the car reading previous reports and when asked what the RN was looking for, the RN responded with: ‘…clues that give me an idea on how, and who, this is…’. The RN was looking for an idea on who this was to prepare for what might be encountered, for example, how progressed illnesses were affecting them or how they lived their life. What was normal for this person, what could be expected. Detecting clues in reports and converting them into useful information was a form of preparation for these meetings and required RNs to take advantage of their professional knowledge toolkit.

RNs' leadership implied prioritising, which entailed the use of professional knowledge. RNs prioritised their work and creating a workday that was effective for them while ensuring quality care. Here were nuances in the data. RNs with more work experience provided more complex reasoning for their prioritisation (e.g., prioritising which older adult to see and when to see them). The RNs in this study organised a schedule before starting their work; however, this schedule was altered several times during the shift. Some RNs based their priorities on what would be the correct time to provide a specific medication; others had a more complex and intertwined approach that accounted for several factors like geography, relationships, knowledge of how the older adult lived, how the previous day had been, what they knew the older adult was doing on a given day and planning around alarms that they knew were coming.

RNs also prioritised what to do during their time with the older adult. RNs used professional knowledge to interpret the dynamics of situations and prioritise accordingly. For example, one RN set aside scheduled tasks to attend to the immediate needs of a distressed next‐of‐kin. Upon arriving at a home, the RN encountered a next‐of‐kin who was clearly distressed about a piece of broken medical equipment. Once reassured that there was no immediate threat to the older adult's safety, the RN shifted attention to the next‐of‐kin. The next‐of‐kin's distress extended beyond the broken equipment itself and reflected concerns about responsibility, uncertainty and the possibility of future crises. The RN listened to the next‐of‐kin, giving them time and space to talk about fears and plans for handling the situation now and in the future. The RN was focused on the next‐of‐kin. Priority one was ensuring the safety of the older adult without this equipment and secondly the next‐of‐kin. None of the planned tasks were completed during this visit. What might initially have appeared to be a technical issue involving broken equipment ultimately became an act of relational care, in which emotional support, attentive listening and the management of uncertainty were as central as the clinical assessment itself.

### 4.4 Leveraging Situations

The overarching theme of leveraging situations suggests that RNs in HHC were combining the three themes in their leadership. RNs were leading older adults, next‐of‐kins and care staff towards common goals, sometimes in subtle ways, while simultaneously performing predefined tasks close to older adults in HHC. RNs were leading close to older adults by leveraging situations they encountered during their workday, which entailed starting from the older adult, adapting to the particular and utilising professional knowledge.

RNs often leveraged situations in subtle ways; for instance, seemingly casual conversations were often turned into something more. During such an everyday conversation regarding the neighbours of the older adult, while administering medications, the RN picked up on a subtle clue from the older adult about a fear of fire alarms. The RN explored further and discovered that the older adult avoided using the stove, like previously assumed, not because of fire risk but because the older adult feared the noise of the fire alarm. The older adult would be unable to turn it off. As a result, she ate premade dinners cold and thought she might be losing weight. The RN addressed the issue on the spot by arranging for the purchase of a microwave oven. The RN contacted next‐of‐kin and provided instructions for them. Back at base, the RN sent messages to the purchaser‐office to request an allocation to follow up on nutritional needs and to add weighing to the older adult's care plan. By adapting to the particular, starting out from the older adult and utilising professional knowledge, the RN leveraged the situation in favour of the shared goal of safe, high‐quality care for the older adult in HHC.

## 5 Discussion

The aim of this study was to explore how RNs lead close to older adults in HHC. The findings provide insights into how RNs were leading close to older adults by leveraging situations they encountered during their workday. A prevalent way for municipalities in Norway to organise and manage HHC is the purchaser–provider model. Tasks based on time allocation can be incompatible with RNs' understanding of care (Petersen et al. 2024), and the organisational characteristics of HHC, such as time margins, have been shown to be predictors of moral distress (Petersen and Melzer 2023). On the one hand, leveraging situations can provide older adults with more individually customised services. On the other hand, it could be disguising some potential consequences of a healthcare system that facilitates task‐orientation through its organisational structure. RNs can face multidimensional problems in their professional work; nevertheless, they are governed to manage them one dimensionally (Vabø 2014a). Going beyond predefined tasks and allocated time to benefit patients seems to be a part of the RNs' daily work in municipal HHC (Brenne et al. 2024; Strandås et al. 2019). Self‐managed multidisciplinary teams could be an alternative to the purchaser–provider model (Eide et al. 2022).

This study explored how RNs' leadership close to older adults is enacted in direct nursing care in municipal HHC. Some findings may resonate with established ideals, such as person‐centred care and holistic nursing practice. However, the analytical focus of this study was not on these perspectives. Several ethical conflicts can arise for RNs in HHC, such as the feeling of being powerless to advocate for the patient (Porr et al. 2024). Leading close to patients involves more than nursing interventions; it can imply daring to challenge the organisation (Claesson et al. 2024), perhaps particularly in the task‐focused organisation of HHC. The findings indicated that RNs' leadership close to older adults could mask some organisational difficulties for HHC to view older adults holistically and place them at the centre. This potentially results in the responsibility of holistic care being placed on individual RNs without organisational support, which may risk contributing to both older adults' vulnerability and moral distress of RNs.

The findings indicated that RNs lead close to older adults by starting from the older adults. The findings in this study indicate an alignment with a person‐centred approach (Fjørtoft et al. 2021; Brenne et al. 2022; Hoglander et al. 2020; Håkansson Eklund et al. 2019). Part of RNs' distinctive work in HHC is providing customised solutions in different homes (Fjørtoft et al. 2021). The RNs concerned themselves with the needs they met, and RNs in this study led by starting from the older adult to adopt an individual and holistic approach. The national code of ethics for RNs includes the provision of holistic care (Norsk Sykepleieforbund 2023). The international code of ethics involves the provisions for person‐centred care (International Council of Nurses 2021). Combined with this study's findings, this indicates that RNs' leadership close to older adults can be a congruent form of leadership, meaning it matches values to actions (Stanley et al. 2022; Northouse 2022). A previous study showed that systematically working in a more person‐centred way could improve RNs' leadership (Lillsjö et al. 2023).

This study showed that RNs lead by adapting to the particular situations they encounter and indicated that RNs in HHC adapt themselves to the older adults they meet. In general, leadership is well known to influence patient safety (Wong et al. 2013; Murray and Cope 2021). Risk prevention in patients' homes can be challenging, for example, managing safety while at the same time respecting the patients' self‐determination (Lekman et al. 2023). Relationships and trust with healthcare workers are vital parts of older adults feeling safe in their own homes (James et al. 2019; Silverglow et al. 2021). Leadership close to patients can demand sensitivity in the moment and involve reflections of the RNs' own behaviour when interacting with patients (Claesson et al. 2024). RNs adapting to each situation and older adults can therefore influence patient safety. Strengthening nursing leadership can be accomplished via targeted educational interventions (Cummings et al. 2021). Therefore, targeted educational interventions for RNs in leadership skills could have a positive effect on older adults' safety at home.

The findings suggest that RNs in HHC utilise their professional knowledge both to prepare their leadership and to lead. HHC involves meetings wherein RNs interact with and observe older adults; in this study, the RNs connected the older adults' realities and wishes, as well as clinical observations, to professional knowledge. Therefore, leadership close to older adults relies on how RNs perceive and interpret situations. Clinical knowledge sensitises RNs to possible issues (Benner et al. 2009), and when guidelines and procedures do not accommodate the complexities in HHC RNs rely on judgement (Brenne et al. 2022). This is in line with the current study, which indicated that RNs apply professional knowledge to interpret the dynamics of situations and prioritise accordingly. RNs in HHC take an active role in negotiating patient service levels, and RNs mediate and can coordinate due to a lack of coordinative mechanisms in the healthcare system (Melby et al. 2018). RNs in HHC act as sentient actors (Vabø 2014b), meaning that their work is based on planning and technical skills while being aware of the realities of older adults. RNs are crucial in assessing needs, following up with advanced care and organising the work in HHC (Fjørtoft et al. 2021), keeping things together and tying up loose ends (Fjørtoft et al. 2021; Melby et al. 2018). A broad foundation of knowledge is essential to perceive and understand complex situations accurately and to anticipate outcomes. Illustrating this complexity, leading close to the older adults implies RNs being multi‐artists (Claesson et al. 2020). Therefore, professional knowledge combined with leadership skills could be considered essential for RNs' leadership close to older adults.

## 6 Limitations

This study explores leadership close to older adults, not management. Nevertheless, management often occurs in parallel with leading. Future studies on leadership close to older adults can seek to bridge these two processes and explore them as co‐dependent. Participant observations make the observer a part of the observations, risking social desirability bias and the Hawthorne effect (Small and Clarco 2022). The observer encountered no examples of a negative or coercive form of leadership in this study and witnessed no misuse of trust. Older adults without the capacity to provide consent were excluded from participation. This might be a limitation because older adults without capacity for consent might add to the diversity of older adults in HHC and are a considerable part of people receiving HHC. Participant observations are influenced by the observer's presence and interpretations (Spradley 1980). However, the RNs were asked to provide input and reflections on these interpretations at the end of the observations. The sample consisted of eight RNs during a limited time. Consequently, transferability beyond this study should be made with caution. However, the findings may reflect the experiences of RNs working under similar conditions.

## 7 Conclusions

This study provides insights into RNs' leadership close to older adults in HHC. The participant observations revealed that this leadership entails leveraging situations within the organisational framework, implying a need for RNs' flexibility and the ability to adapt to the prerequisites in this sector to provide good and safe care. These findings indicate it is time for organisations to align the care for older adults and for those who provide and lead the care. The findings illustrate how RNs enact leadership close to older adults through situated and adaptive practices in direct nursing care. The adaptive leadership practices of RNs might affect the quality of care. Providing RNs with prerequisites to lead close to older adults, through means such as guidelines and educational efforts, might contribute to the long‐term sustainability of municipal HCC.

## Author Contributions

All authors listed have made a substantial, direct and intellectual contribution to the study. K.J. was the main supervisor of the study and generated the idea for the study. K.J., L.‐L.J. and T.S. designed the study; T.S. performed the data collection with supervision by L.H.S.; T.S. performed the data analysis, structured the results and created the intellectual content in the discussion; and T.S. was the writer of the manuscript. K.J., L.‐L.J. and L.H.S. provided critical revision and supervision. All authors read and approved the final manuscript.

## Funding

The authors have nothing to report.

## Ethics Statement

This study from Nord University was assessed by the Regional Committee for Medical Research Ethics Northern Norway, REK North (D. no: 753491), and approval was waived according to national legislation, §2 and §9 of the Health Research Act (Act on Medical and Health Research 2008). This study adheres to good research practices as outlined in the guidelines from the Helsinki declaration (World Medical Association 2024). The study is registered (D. no: 478281) at the Norwegian Agency for Shared Services in Education and Research (SIKT).

## Conflicts of Interest

The authors declare no conflicts of interest.

## Data Availability

The data that support the findings of this study are available from the corresponding author upon reasonable request.
